# Supplementary material for: Gold nanocrystal-mediated sliding of doublet DNA origami filaments
Source: Nat Commun. 2018 Apr 13;9:1454. doi: 10.1038/s41467-018-03882-w (PMC5899135; doi:10.1038/s41467-018-03882-w)
Supplement: Supplementary file 1 — Supplementary Information [file 41467_2018_3882_MOESM1_ESM.pdf]

Gold nanocrystal-mediated sliding of doublet DNA origami filaments

Urban et al.

Supplementary Figures

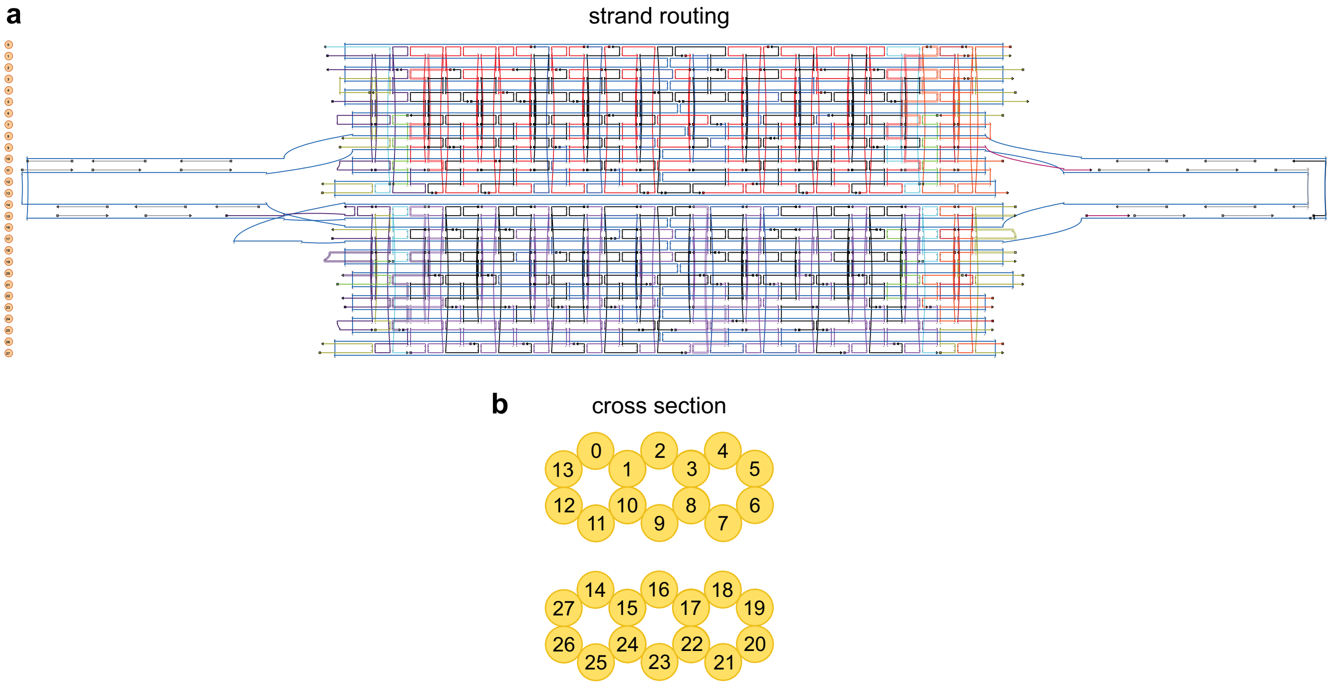

Supplementary Figure 1 DNA origami design details. **a**) Strand routing diagram of the DNA origami structure. The two 14-helix bundles (14 hb) are linked by four single-stranded DNA segments of ~200 nucleotides. **b**) Details of the 14 hb design. The DNA origami structure was designed using caDNAno software. The DNA origami filaments consist of 2 ×14 helices arranged in a 'honeycomb' lattice. To prevent the aggregation of the DNA origami, six thymine bases were added to the respective staple strands at the edge of the origami.

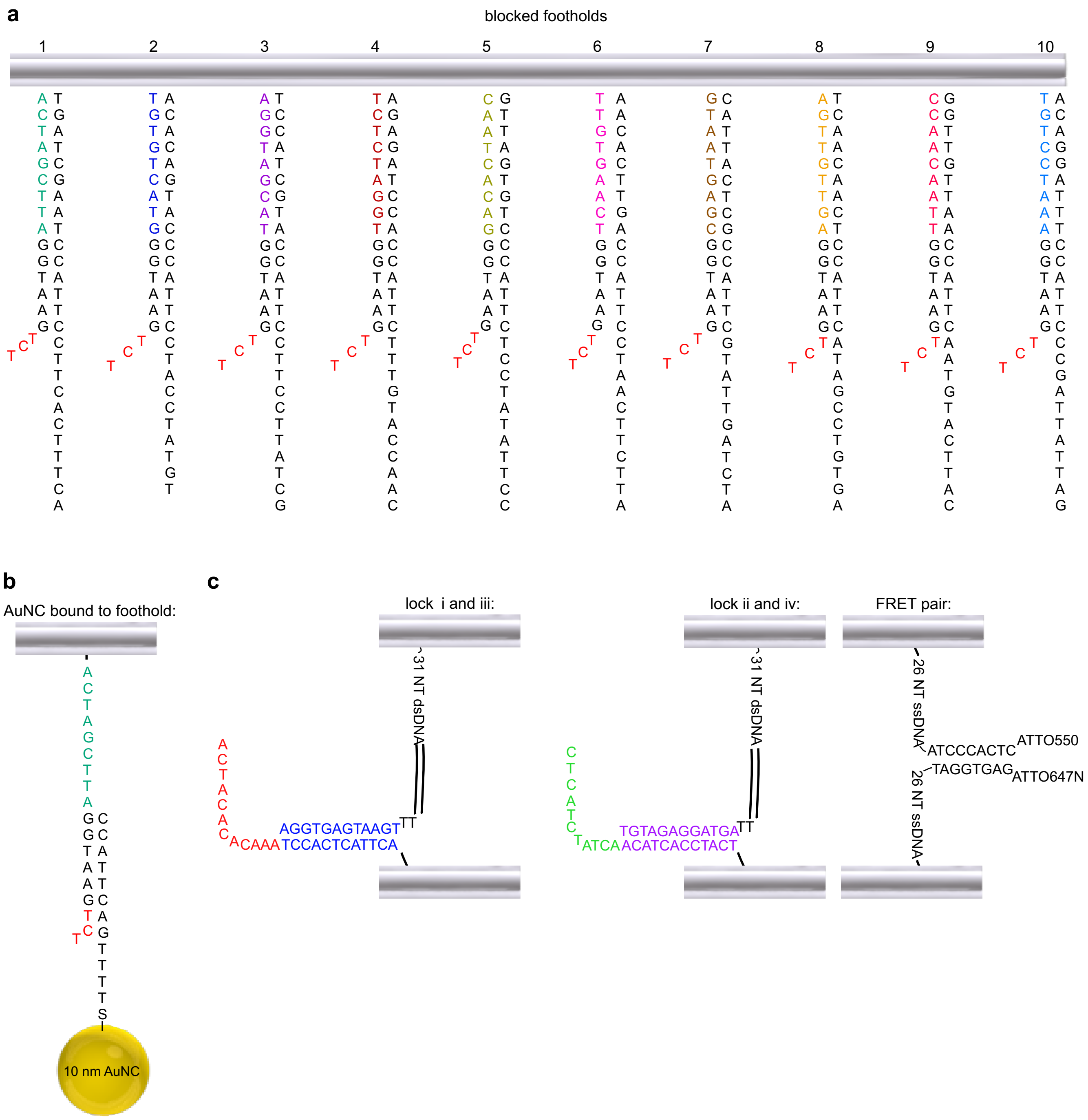

Supplementary Figure 2 DNA sequence details of the origami filament for sliding. **a**) Sequences of the ten different footholds in a blocked state. **b**) An 8 nt foot sequence is bound on the surface of the AuNC via a thiol modification and a linker of four thymidine nucleotides. **c**) Design details of the locks and the fluorescently modified DNA strands.

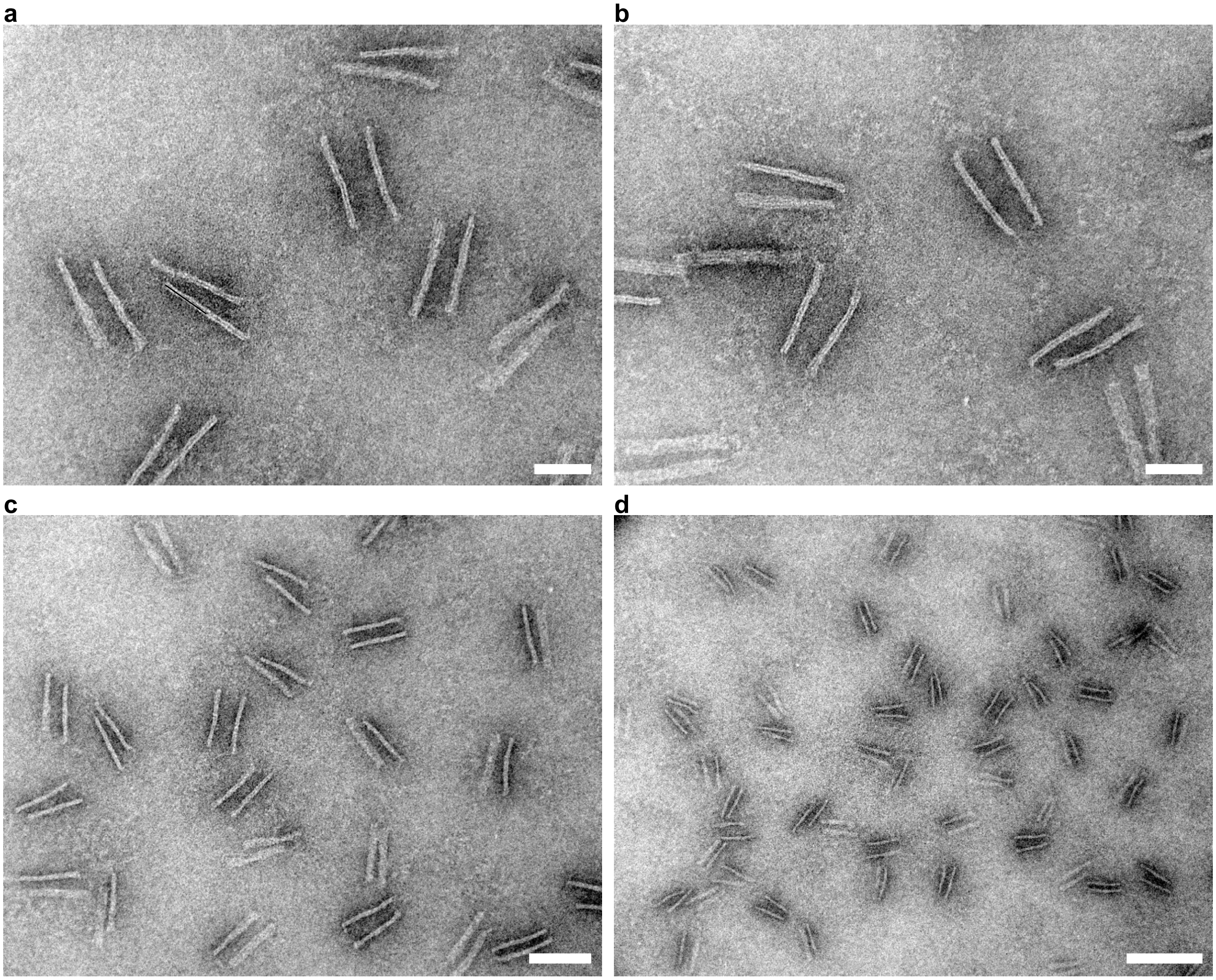

Supplementary Figure 3 TEM images of the DNA origami filaments. Scale bars: **a,b**) 50 nm, **c**)100 nm and **d**) 200 nm

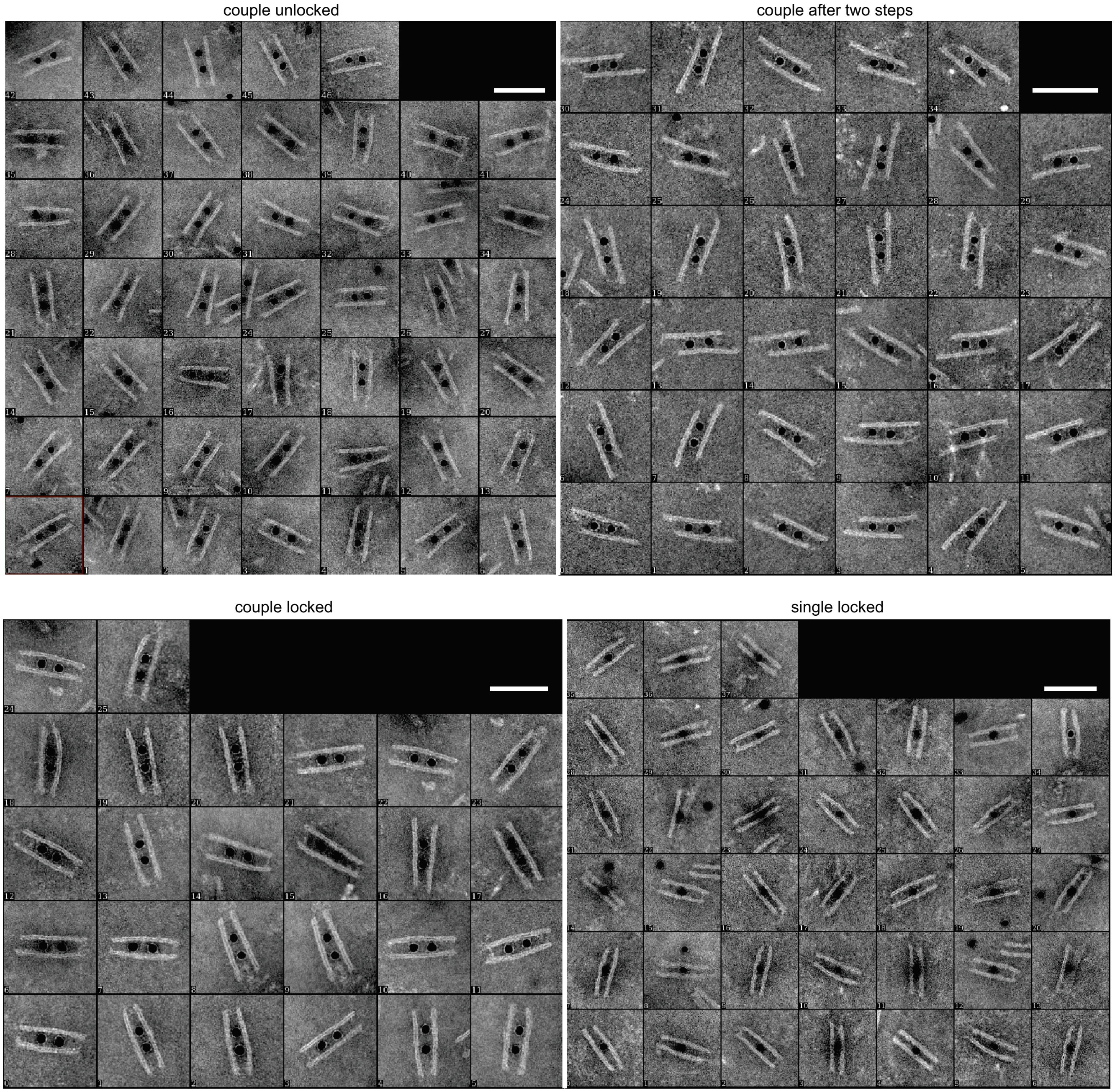

Supplementary Figure 4 Structure libraries for average TEM images. Scale bars: 100 nm

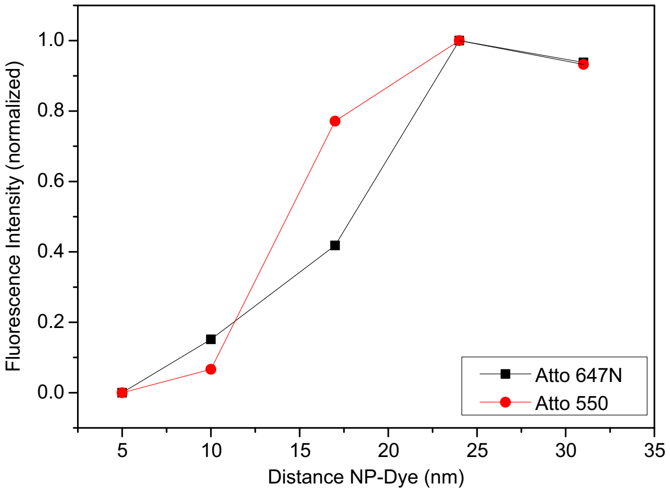

Supplementary Figure 5 Measured quenching curves. For measurements of Atto 550, the dye was excited at 530 nm and emission spectra were recorded for five states. For measurements of Atto 647N, the dye was excited at 600 nm and emission spectra were recorded. For individual fluorescence spectrum measurements, the sample at the initial state was divided into 5 copies. All of the copies had a volume of 120 μL and respective strands were added to drive the individual systems to their designated states. To keep the system concentration constant, equal volume of H_2O was added, if no DNA strands were added. Two additions were conducted for all of the 5 samples. After each time of the addition, the samples were incubated at room temperature for about 2 h to ensure that they reached equilibrium. Fluorescence intensities are normalized for comparison.

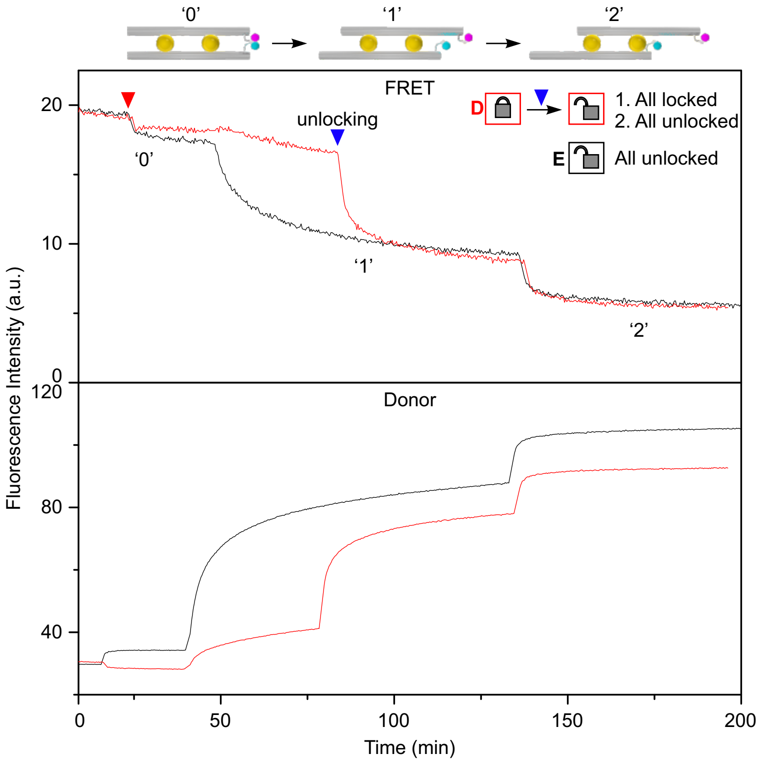

Supplementary Figure 6 Relative sliding in the presence of DNA side locks. FRET and donor fluorescence signals monitored during sliding for samples with four and zero locks. The red arrow indicates the starting point of the experiments. In sample D all locks were locked at first. After the addition of sliding trigger DNA strands, a clear influence of the locks on the sliding behavior can be observed. During the sliding process from ‘0’ to ‘1’ the unlocking strands were added. An abrupt response can be observed in the FRET signal. The sample approaches the behavior of the sample E, which was unlocked from the beginning. A subsequent step from ‘1’ to ‘2’ shows comparable optical responses for both samples. This finding shows that sliding is inhibited by the locks and as soon as the locks are opened, sliding occurs. The kinetics is faster because the rearrangements to activate the footholds on the tracks have started already earlier. Sample E and D show differences in the fluorescence intensities of the final state in the donor measurements. This might be attributed to a lower sliding yield in the walking scheme of sample D. Furthermore, this result shows that the AuNCs remain functional and perform their dedicated task as soon as the locks are opened.

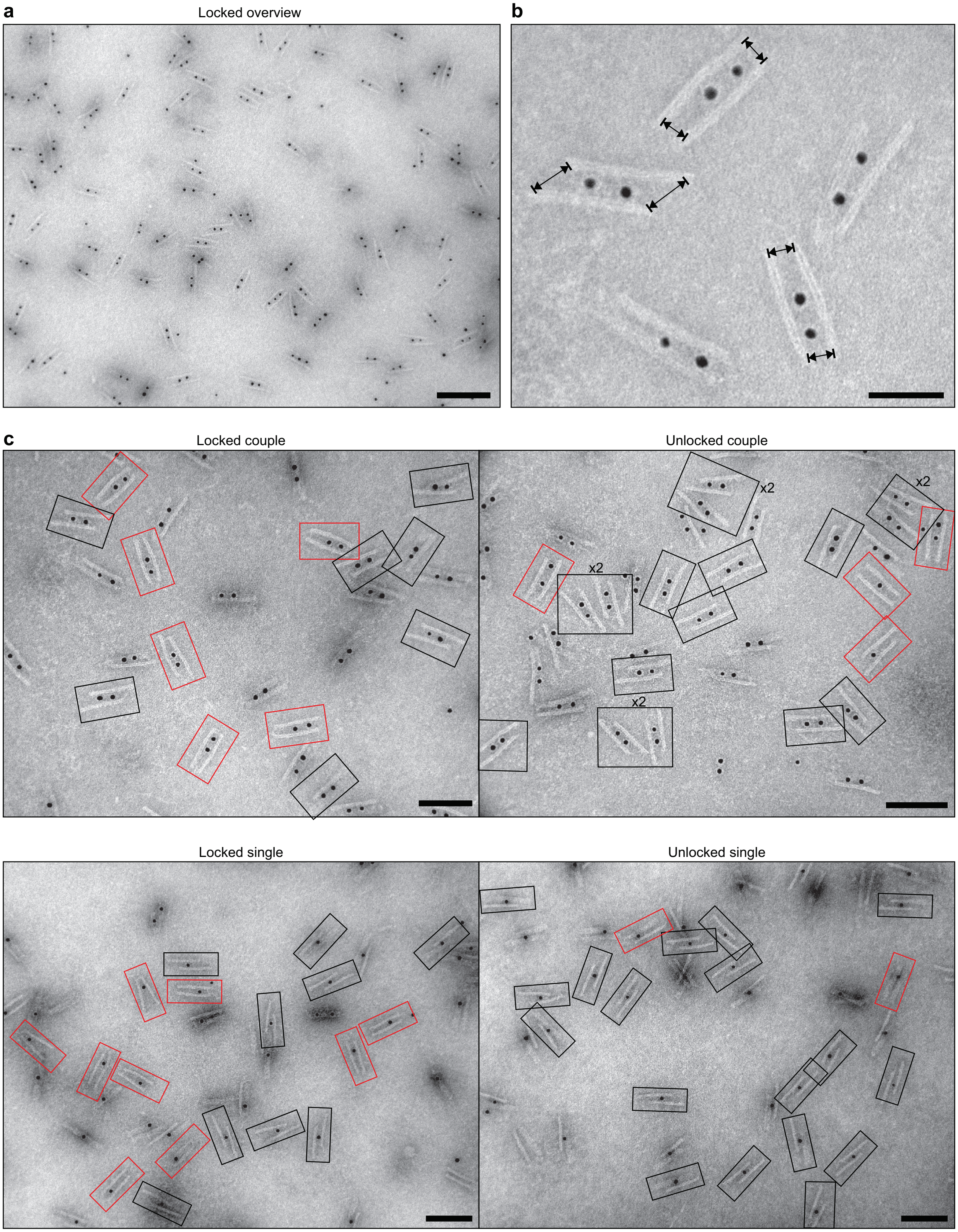

Supplementary Figure 7 Exemplary TEM images of the sliding systems in the locking experiments. **a**) Overview image of the locked AuNC couple after two sliding steps. Statistical analysis of the assembly yield of initial structures showed 87% yield. After two steps of sliding 85% of the structures reached the slided state. Scale bar: 200 nm. **b**) Distance measurements for Fig. 4d in the main text. Scale bar: 50 nm. **c**) Classification of the structures after two sliding steps. Structures were qualitatively divided in a group that slided (black boxes) and a group that did not slide (red boxes). Samples for TEM analysis were taken 80 minutes after addition of sliding fuels (see Fig. 4, main text). Scale bars: 100 nm.

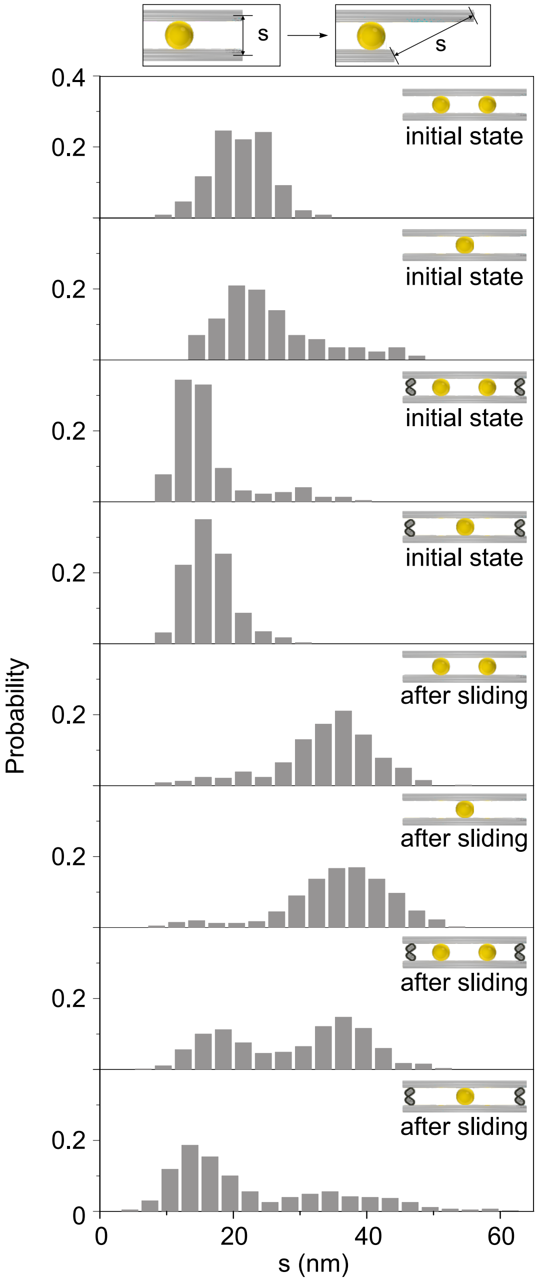

Supplementary Figure 8 Histograms of the end-to-end distance (s) of the filaments at the initial state and after two sliding steps. Inset images describe the locking states of the structures.

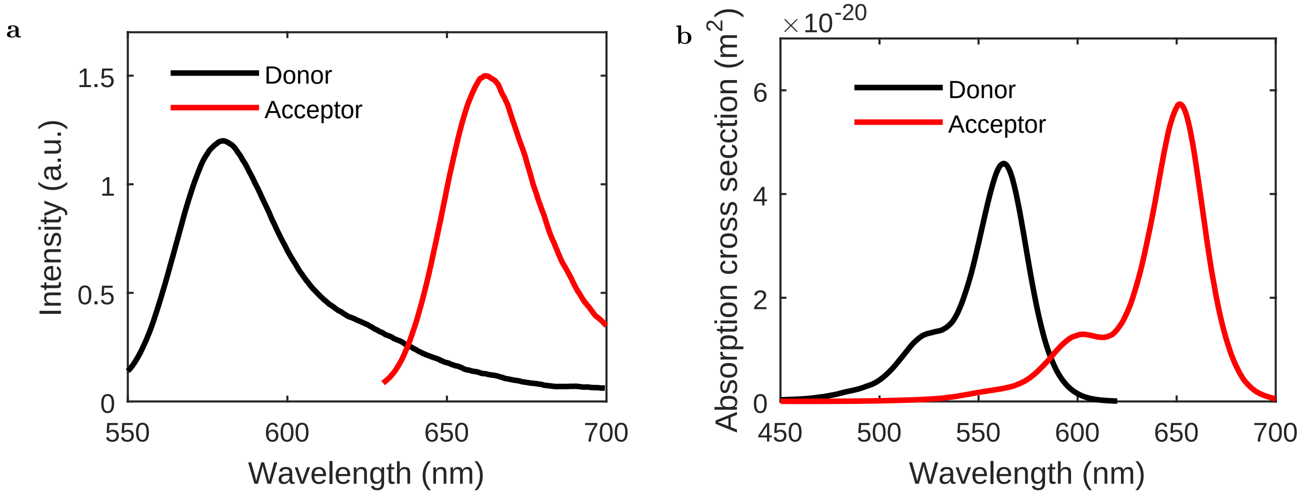

Supplementary Figure 9 Dye properties. **a**) Measured intrinsic fluorescence spectra of donor and acceptor (not normalized) attached to the DNA structure. The peak wavelengths are 578 nm for the donor and 663 nm for the acceptor. **b**) Absorption cross sections of donor and acceptor attached to the DNA origami, obtained from measured excitation spectra. The measured curves provided here are used as the basis for all theoretical calculations (Note that the absorption cross section of the donor is actually not needed for the calculations and only plotted here for the sake of completeness).

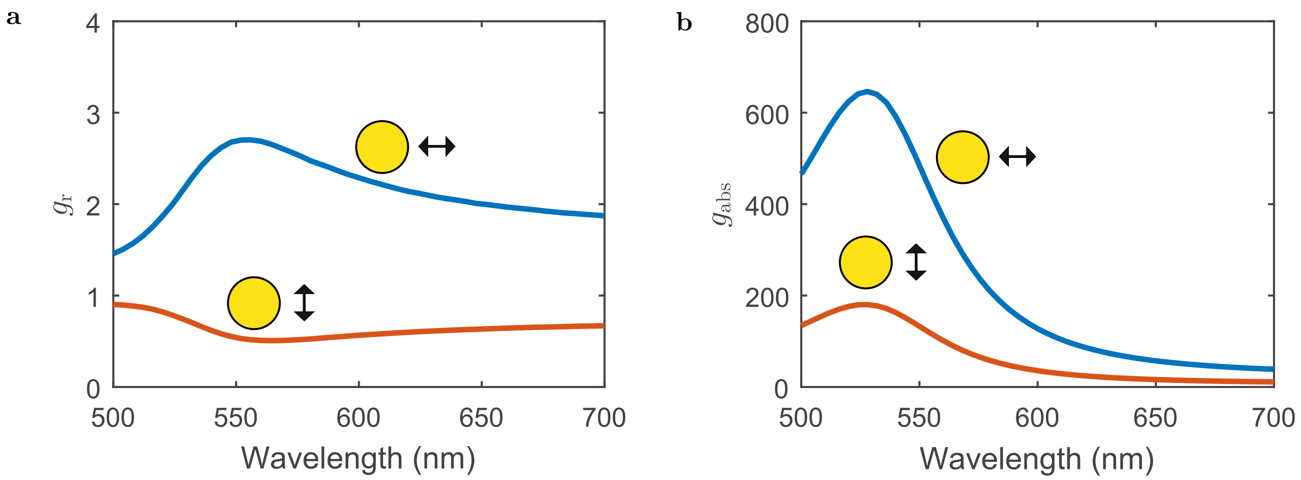

Supplementary Figure 10 Simulation results for g_r and g_{abs} at an exemplary distance of 5 nm between the dipole and the nanoparticle. **a**) Enhancement factor for the radiative rate $g_r(\omega)$ plotted for two different dipole orientations. **b**) Energy transfer factor $g_{abs}(\omega)$ for the same dipole orientations. The enhancement factor for the radiative rate exhibits a peak or a dip at around 550 nm, depending on the dipole orientation, while the energy transfer factor exhibits a peak at around 530 nm for both dipole orientations.

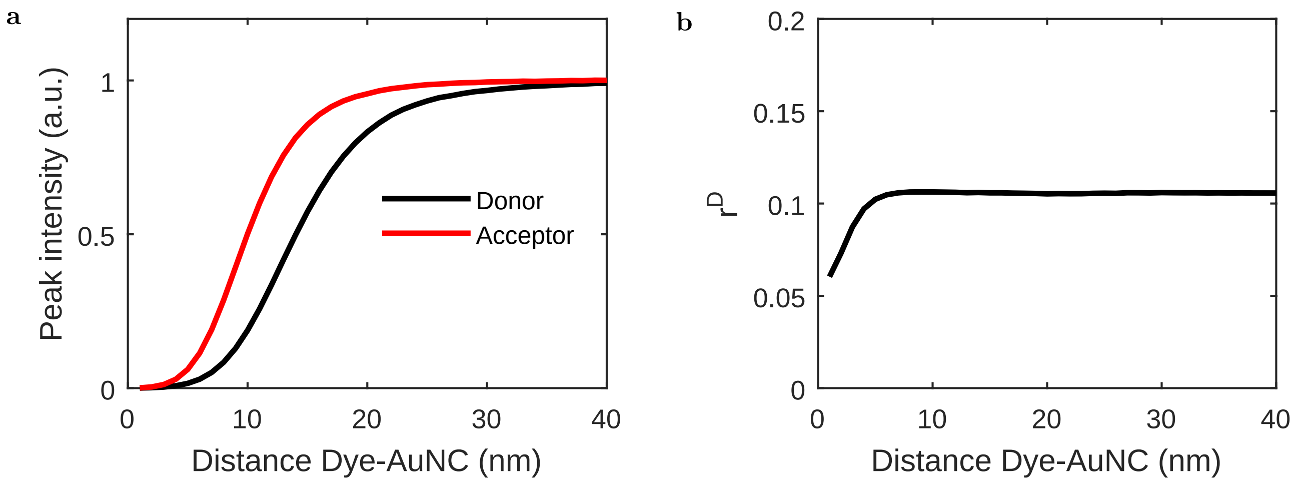

Supplementary Figure 11 Results of the quenching simulations and calculated distance dependence of the factor r^{D} defined in (33). **a**) Donor and acceptor peak intensities as a function of distance between the dye and the nanoparticle, averaged over different dye orientations. The intensities are normalized to the peak intensities of the intrinsic dye spectra. The peak wavelengths are 578 nm for the donor and 663 nm for the acceptor. It was found that the peak wavelengths with and without nanoparticle are identical. **b**) Ratio of the donor intensity at the acceptor peak wavelength and the donor intensity at the donor peak wavelength, plotted as a function of the distance between the donor and the nanoparticle. This result is used to justify that Eq. (33) together with a value of $r^{\mathrm{D}} = 0.106$ can be used to extract the donor and acceptor contributions from experimental data.

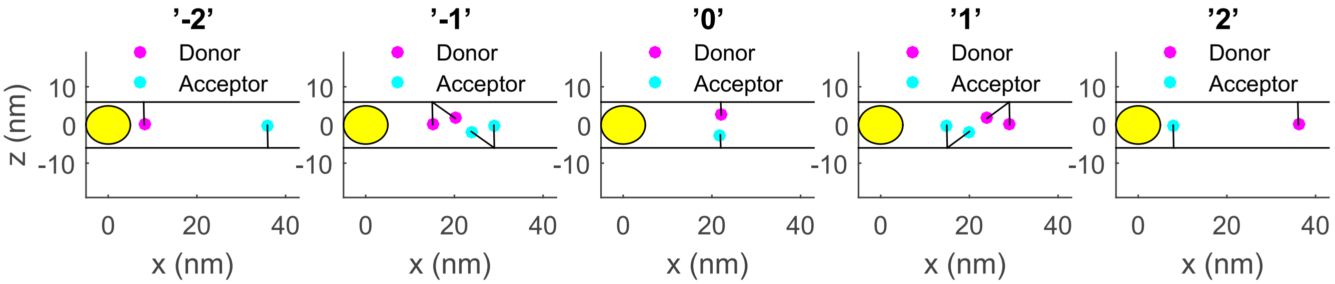

Supplementary Figure 12 Dye positions during the individual sliding states. There are two parallel DNA origami filaments with a distance of 12 nm. The dyes are connected to the filaments by single DNA strands of length 6 nm. The dye positions that are used in the calculation are drawn into a coordinate system with the origin being chosen as the center of the nanoparticle. The coordinates can be found in Supplementary Table 2. The '-2' and '2' sliding states were described with the DNA strands standing perpendicular to the filaments. This configuration is referred to as unbound state. For the '0' sliding states, it was assumed that the dyes form a bound state with a distance of 5 nm between the donor and the acceptor, due to a transient binding of the single DNA strands. The '-1' and '1' sliding state were modeled as a 50%:50% distribution of dye pairs in a bound state and dye pairs in an unbound state.

Supplementary Tables

Supplementary Table 1 Samples added to drive the sliding for real time fluorescence detection in Fig. 3 (main text). The blocking and removal strands were prepared at a concentration of 200 μM in H_2O. Generally, small volumes but high concentrations of the blocking and removal DNA strands were used for driving the AuNCs to reduce the dilution effect. After the entire process, the total volume increase was 22 μL (18%). The raw data were dilution corrected by calculation of the signal reduction through dilution and addition of this value.

Step	Strands added	Volume added per strand
'0' → '-1'	3,7,$\overline{5}$,$\overline{9}$	0.25 μL
'-1'→ '-2'	4,8,$\overline{6}$,$\overline{10}$	0.25 μL
'-2'→ '-1'	6,10,$\overline{4}$,$\overline{8}$	0.5 μL
'-1'→ '0'	5,9,$\overline{3}$,$\overline{7}$	0.5 μL
'0'→ '1'	4,8,$\overline{2}$,$\overline{6}$	0.75 μL
'1'→ '2'	3,7,$\overline{1}$,$\overline{5}$	0.75 μL
'2'→ '1'	1,5,$\overline{3}$,$\overline{7}$	1.25 μL
'1'→ '0'	2,6,$\overline{4}$,$\overline{8}$	1.25 μL

Supplementary Table 2 Dye positions as drawn in Supplementary Figure 12. For each sliding state, the table lists the sliding displacement, the coordinates of donor and acceptor, the distance of the individual dye to the surface of the nanoparticle (labeled as Dye-AuNC) and the distance between the donor and the acceptor (labeled as A-D). All distances are given in nm. The '-1' and '1' sliding states are special in a sense that for them, it is assumed that there is a 50%:50% distribution of two different dye configurations.

**State**	**Displacement**		**Donor**			**Acceptor**			**D-A**
			x	z	Dye-AuNC	x	z	Dye-AuNC	
**-2**	**-28**		-2.0	0.0	8.0	26.0	0.0	36.0	28.0
**-1**	**-14**	50%	5.0	0.0	15.0	19.0	0.0	29.0	14.0
		50%	10.1	1.6	20.2	13.9	-1.6	24.0	5.0
**0**	**0**		12.0	2.5	22.1	12.0	-2.5	22.1	5.0
**1**	**14**	50%	19.0	0.0	29.0	5.0	0.0	15.0	14.0
		50%	13.9	1.6	24.0	10.1	-1.6	20.2	5.0
**2**	**28**		26.0	0.0	36.0	-2.0	0.0	8.0	28.0

Supplementary Table 3 Samples added to drive the sliding in Fig. 4 (main text). The raw data were dilution corrected by calculation of the signal reduction through dilution and addition of this value.

Step	Strands added	Volume added per strand
locking	A: lock lock 1, lock lock 2<br>B: lock lock 1, unlock lock 2<br>C: unlock lock 1, unlock lock 2	0.25 μL
'0'→ '1'	4,8,$\overline{2}$,$\overline{6}$	0.5 μL
'1'→ '2'	3,7,$\overline{1}$,$\overline{5}$	0.5 μL

Supplementary Notes

Supplementary Note 1: Spectral correction of the experimental data

Fluorescence spectra measured in the spectral-aquisition mode were spectrally corrected using a reference light source. To correct data measured in the time-scan acquisition mode, the fluorescence signal measured in the spectral-acquisition mode were divided by the signal in time-scan acquisition mode. A correction factor of 0.28 was found for 578 nm and a factor of 1.395 was found for 663 nm. The raw data from time-scan acquisition measurements with multiplied with the respective values.

Supplementary Note 2: Theory

The fluorescence curves in Fig. 3c have been obtained under the assumption that we can treat the dyes as electric point dipoles in order to describe the electromagnetic interaction between the dyes and the nanoparticles. In section **Equations**, we derive the governing equations for our model, resulting in the fluorescence curves of donor and acceptor. In section **Simulation details**, we present all input parameters that were used as basis for our calculations, give details on the numerical evaluation, and discuss intermediate results.

Equations

Our model takes into account two effects: quenching of the two dyes by the nanoparticles and FRET from the donor to the acceptor. The quenching equations were taken from Ref. [1], which provides a theoretical model to calculate the emission spectrum of a dye molecule coupled to a plasmonic resonator. We extended this model in order to describe a system where both quenching and FRET are present. In this section, we first revisit the derivation of the quenching equations and then show how the intermediate steps of the derivation have been modified.

Quenching

In the low excitation regime, the fluorescence rate $\gamma_{\mathrm{fl}}^{\mathrm{x}}$ of a dye is given as product of its quantum yield q^{x} and excitation rate $\gamma_{\mathrm{exc}}^{\mathrm{x}}$:[2]

$$\gamma_{\mathrm{fl}}^{\mathrm{x}} = q^{\mathrm{x}} \gamma_{\mathrm{exc}}^{\mathrm{x}}. \tag{1}$$

Here, the superscript $\mathrm{x} = \mathrm{D,A}$ is introduced to label quantities of the donor and acceptor, respectively, which explicitly depend on intrinsic dye properties. For the FRET equations, the subscript will be used to distinguish between the donor and the acceptor. In general, the quantum yield of a dye is defined as the ratio of its radiative decay rate $\gamma_{\mathrm{r}}^{\mathrm{x}}$ and its total decay rate γ^{x}:[2]

$$q^{\mathrm{x}} = \frac{\gamma_{\mathrm{r}}^{\mathrm{x}}}{\gamma^{\mathrm{x}}}. \tag{2}$$

From knowing the fluorescence rate and the emission frequency of the dye, it is possible to calculate the time-averaged power $P_{\mathrm{fl}}^{\mathrm{x}}$ that is emitted by the dye. The time-averaged power is given in W, while the fluorescence rate is given in photons/s. Both are connected by

$$P_{\mathrm{fl}}^{\mathrm{x}} = \hbar \omega_{\mathrm{fl}}^{\mathrm{x}} \gamma_{\mathrm{fl}}^{\mathrm{x}}, \tag{3}$$

where $\hbar \omega_{\mathrm{fl}}^{\mathrm{x}}$ is the energy of one emitted photon. If the dye does not emit at one single frequency, but over a spectrum $F^{\mathrm{x}}(\omega)$, $\hbar \omega_{\mathrm{fl}}^{\mathrm{x}}$ becomes the average energy of emitted photons. In the following, we show how this average photon energy can be calculated from the fluorescence spectrum $F^{\mathrm{x}}(\omega)$. Let us consider the time-averaged power emitted by the dye, which is given by

$$P_{\mathrm{fl}}^{\mathrm{x}} = \int_0^\infty F^{\mathrm{x}}(\omega) \mathrm{d}\omega. \tag{4}$$

This allows to express the fluorescence spectrum as the product of the time-averaged power $P_{\mathrm{fl}}^{\mathrm{x}}$ and an integral-normalized version $f^{\mathrm{x}}(\omega)$ of the fluorescence spectrum:

$$F^{\mathrm{x}}(\omega) = P_{\mathrm{fl}}^{\mathrm{x}} f^{\mathrm{x}}(\omega). \tag{5}$$

In this case, integral-normalized means that $\int_0^\infty f^{\mathrm{x}}(\omega) \mathrm{d}\omega = 1$. Since the fluorescence rate $\gamma_{\mathrm{fl}}^{\mathrm{x}}$ equals the number of photons emitted per second, it can be obtained by integrating over the spectral photon density, which is defined as spectral power density divided by energy per photon, $F^{\mathrm{x}}(\omega)/\hbar\omega$. Hence, we obtain

$$\gamma_{\mathrm{fl}}^{\mathrm{x}} = \int_0^\infty \frac{F^{\mathrm{x}}(\omega)}{\hbar \omega} \mathrm{d}\omega. \tag{6}$$

Combining Eq. (4), (5), and (6) gives an expression for the average photon energy:

$$\hbar\omega_{\text{fl}}^{\text{x}} = \frac{P_{\text{fl}}^{\text{x}}}{\gamma_{\text{fl}}^{\text{x}}} = \frac{1}{\int_0^{\infty} \frac{f^{\text{x}}(\omega)}{\hbar\omega} \text{d}\omega}. \tag{7}$$

Inserting Eq. (3) into Eq. (5) allows to express the fluorescence spectrum as a function of the fluorescence rate, which is connected to the excitation rate via Eq. (1). This yields:

$$F^{\text{x}}(\omega) = \hbar\omega_{\text{fl}}^{\text{x}} f^{\text{x}}(\omega)\gamma_{\text{fl}} = \hbar\omega_{\text{fl}}^{\text{x}} q^{\text{x}} f^{\text{x}}(\omega)\gamma_{\text{exc}}. \tag{8}$$

With this equation, it is now possible to formally determine the influence of a nanoparticle on the emission spectrum of a dye. Dividing the emission spectrum $F^{\text{x}}(\omega)$ of a dye in the vicinity of a nanoparticle by the emission spectrum $F_0^{\text{x}}(\omega)$ of the dye without the nanoparticle results in

$$\frac{F^{\text{x}}(\omega)}{F_0^{\text{x}}(\omega)} = \underbrace{\frac{\hbar\omega_{\text{fl}}^{\text{x}} q^{\text{x}} f^{\text{x}}(\omega)}{\hbar\omega_{\text{fl},0}^{\text{x}} q_0^{\text{x}} f_0^{\text{x}}(\omega)}}_{g_{\text{em}}^{\text{x}}(\omega)} \underbrace{\frac{\gamma_{\text{exc}}(\omega_{\text{exc}}^{\text{x}})}{\gamma_{\text{exc},0}(\omega_{\text{exc}}^{\text{x}})}}_{g_{\text{exc}}(\omega_{\text{exc}}^{\text{x}})}. \tag{9}$$

Here, the index 0 is used to label quantities in the absence of the nanoparticle and no index is used to label quantities that are influenced by the nanoparticle. Equation (9) shows that the influence of the nanoparticle can be split into two parts: the emission enhancement $g_{\text{em}}^{\text{x}}(\omega)$, and the excitation enhancement $g_{\text{exc}}(\omega_{\text{exc}}^{\text{x}})$.[1] The argument $\omega_{\text{exc}}^{\text{x}}$ is used to emphasize that the excitation process happens at a different frequency than the emission process.

Excitation enhancement. The excitation enhancement can be calculated as[1]

$$g_{\text{exc}}(\omega_{\text{exc}}^{\text{x}}) = \frac{\gamma_{\text{exc}}(\omega_{\text{exc}}^{\text{x}})}{\gamma_{\text{exc},0}(\omega_{\text{exc}}^{\text{x}})} = \frac{|\mathbf{n}\cdot\mathbf{E}_{\text{exc}}(\mathbf{r}_0)|^2}{|\mathbf{n}\cdot\mathbf{E}_{\text{exc},0}(\mathbf{r}_0)|^2}, \tag{10}$$

where $\mathbf{n}$ is a unit vector that describes the orientation of the dipole, while $\mathbf{E}_{\text{exc}}(\mathbf{r}_0)$ and $\mathbf{E}_{\text{exc},0}(\mathbf{r}_0)$ are the incident electric fields at the position of the dipole $\mathbf{r}_0$ with and without the nanoparticle, respectively.

Emission enhancement. In the emission enhancement, q_0^{x} and $f_0^{\text{x}}(\omega)$ are intrinsic properties of the dye, while q^{x} and $f^{\text{x}}(\omega)$ are properties that are influenced by the nanoparticle. The intrinsic quantum yield of the dye is defined by Eq. (2) as the ratio of the radiative decay rate over the total decay rate, which gives $q_0^{\text{x}} = \gamma_{\text{r},0}^{\text{x}}/\gamma_0^{\text{x}} = \gamma_{\text{r},0}^{\text{x}}/(\gamma_{\text{r},0}^{\text{x}} + \gamma_{\text{nr},0}^{\text{x}})$, with $\gamma_{\text{nr},0}^{\text{x}}$ being the intrinsic nonradiative decay rate. With nanoparticle, the quantum yield becomes[2]

$$q^{\text{x}} = \frac{\gamma_{\text{r}}^{\text{x}}}{\gamma_{\text{r}}^{\text{x}} + \gamma_{\text{abs}}^{\text{x}} + \gamma_{\text{nr}}^{\text{x}}}, \tag{11}$$

where $\gamma_{\text{r}}^{\text{x}}$, $\gamma_{\text{abs}}^{\text{x}}$, and $\gamma_{\text{nr}}^{\text{x}}$ are the radiative decay rate, the rate of energy transfer to the nanoparticle and the nonradiative decay rate in the presence of the nanoparticle, respectively. These rates will now be expressed as a function of the intrinsic radiative decay rate $\gamma_{\text{r}}^{\text{x}}$. It is reasonable to assume that the nonradiative decay inside the dye is not influenced by the nanoparticle ($\gamma_{\text{nr}}^{\text{x}} = \gamma_{\text{nr},0}^{\text{x}}$), which yields[2]

$$\gamma_{\text{nr}}^{\text{x}} = \gamma_{\text{r},0}^{\text{x}} \frac{1 - q_0^{\text{x}}}{q_0^{\text{x}}}. \tag{12}$$

The radiative decay rate with nanoparticle is given as[1]

$$\gamma_{\text{r}}^{\text{x}} = \gamma_{\text{r},0}^{\text{x}} \int_0^{\infty} f_0^{\text{x}}(\omega) g_{\text{r}}(\omega) \text{d}\omega, \tag{13}$$

with $g_{\text{r}}(\omega)$ being the enhancement factor for the radiative rate, defined as

$$g_{\text{r}}(\omega) = \frac{P_{\text{rad}}(\omega)}{P_0(\omega)}. \tag{14}$$

Here, $P_0(\omega)$ is the power radiated by an isolated oscillating dipole and $P_{\text{rad}}(\omega)$ is the power radiated by a combination of dipole and nanoparticle. The rate of energy transfer to the nanoparticle is[1]

$$\gamma_{\text{abs}}^{\text{x}} = \gamma_{\text{r},0}^{\text{x}} \int_0^{\infty} f_0^{\text{x}}(\omega) g_{\text{abs}}(\omega) \text{d}\omega, \tag{15}$$

with the energy transfer factor

$$g_{\mathrm{abs}}(\omega)=\frac{P_{\mathrm{abs}}(\omega)}{P_0(\omega)}, \tag{16}$$

where $P_{\mathrm{abs}}(\omega)$ is the power absorbed by the nanoparticle. Note that the factors $g_{\mathrm{abs}}(\omega)$ and $g_{\mathrm{r}}(\omega)$ depend on the orientation of the emitting dipole.

Using the definition of $g_{\mathrm{r}}(\omega)$, the integral-normalized emission spectrum $f^{\mathrm{x}}(\omega)$ in the presence of the nanoparticle can be calculated as follows: The emission spectrum with nanoparticle is proportional to the intrinsic emission spectrum multiplied by the enhancement factor for the radiative rate $g_{\mathrm{r}}(\omega)$, which gives $f^{\mathrm{x}}(\omega)\propto g_{\mathrm{r}}(\omega)f_0^{\mathrm{x}}(\omega)$. Applying the normalization condition $\int_0^\infty f^{\mathrm{x}}(\omega)\mathrm{d}\omega=1$ then leads to

$$f^{\mathrm{x}}(\omega)=\frac{g_{\mathrm{r}}(\omega)f_0^{\mathrm{x}}(\omega)}{\int_0^\infty f_0^{\mathrm{x}}(\omega)g_{\mathrm{r}}(\omega)\mathrm{d}\omega}. \tag{17}$$

The last quantities that are needed for calculating the emission enhancement are the average photon energies $\hbar\omega_{\mathrm{fl},0}^{\mathrm{x}}$ and $\hbar\omega_{\mathrm{fl}}^{\mathrm{x}}$ with and without nanoparticle, respectively, which can in principle be calculated by inserting $f_0^{\mathrm{x}}(\omega)$ and $f^{\mathrm{x}}(\omega)$ into Eq. (7); however, for the sake of simplicity, it will be assumed that the influence of the nanoparticle on the average photon energies can be neglected.

Final quenching equation. Inserting the expressions for q^{x} and $f^{\mathrm{x}}(\omega)$ into Eq. (8), normalizing to the peak of the intrinsic dye spectrum $F_0^{\mathrm{x}}(\omega_{\mathrm{max}})$, using the definition of the excitation enhancement in Eq. (10), and neglecting the change of the average photon energy ($\hbar\omega_{\mathrm{fl}}^{\mathrm{x}}=\hbar\omega_{\mathrm{fl},0}^{\mathrm{x}}$) yields the final quenching equation

$$\frac{F^{\mathrm{x}}(\omega)}{F_0^{\mathrm{x}}(\omega_{\mathrm{max}})}=\frac{g_{\mathrm{r}}(\omega)f_0^{\mathrm{x}}(\omega)}{\int_0^\infty f_0^{\mathrm{x}}(\omega)g_{\mathrm{r}}(\omega)\mathrm{d}\omega+\int_0^\infty f_0^{\mathrm{x}}(\omega)g_{\mathrm{abs}}(\omega)\mathrm{d}\omega+(1-q_0^{\mathrm{x}})/q_0^{\mathrm{x}}}\,\frac{g_{\mathrm{exc}}(\omega_{\mathrm{exc}}^{\mathrm{x}})}{q_0^{\mathrm{x}}f_0^{\mathrm{x}}(\omega_{\mathrm{max}})}. \tag{18}$$

In this equation, q_0^{x} and $f_0^{\mathrm{x}}(\omega)$ are intrinsic properties of the dye and $g_{\mathrm{r}}(\omega)$, $g_{\mathrm{abs}}(\omega)$, and $g_{\mathrm{exc}}(\omega_{\mathrm{exc}}^{\mathrm{x}})$ can be obtained by numerical simulations. Note that this equation formally resembles the equations provided in Ref. [1].

FRET

The FRET rate between a donor D and an acceptor A separated by a distance R is given by the well-known equation[3]

$$\gamma_{\mathrm{FRET}}=\gamma_{\mathrm{r},0}^{\mathrm{D}}\frac{1}{q_0^{\mathrm{D}}}\left(\frac{R_0}{R}\right)^6. \tag{19}$$

Here, the term q_0^{D} arises due to using the intrinsic radiative decay rate $\gamma_{\mathrm{r},0}^{\mathrm{D}}$ instead of the intrinsic total decay rate γ_0^{D}, which is commonly used in most textbooks. R_0 is the Förster radius, which is defined as[3]

$$R_0^6=\frac{q_0^{\mathrm{D}}9c^4\kappa^2}{8\pi}\int_0^\infty\frac{f_0^{\mathrm{D}}(\omega)\sigma_{\mathrm{A}}(\omega)}{n^4(\omega)\omega^4}\mathrm{d}\omega, \tag{20}$$

where $n(\omega)$ is the refractive index of the surrounding medium; $\kappa^2=[\mathbf{n}_{\mathrm{A}}\cdot\mathbf{n}_{\mathrm{D}}-3(\mathbf{n}_{\mathrm{R}}\cdot\mathbf{n}_{\mathrm{D}})(\mathbf{n}_{\mathrm{R}}\cdot\mathbf{n}_{\mathrm{A}})]^2$ describes the orientations of the dipoles, with $\mathbf{n}_{\mathrm{D}}$ and $\mathbf{n}_{\mathrm{A}}$ as unit vectors in the direction of the donor and acceptor dipole, while $\mathbf{n}_{\mathrm{R}}$ is a unit vector pointing from the donor to the acceptor position; $\sigma_{\mathrm{A}}(\omega)$ is the absorption cross section of the acceptor.

In Eq.(19), it is assumed that the dyes are located in a homogeneous environment and a possible influence of the nanoparticle on the FRET rate can be ignored. When including this influence, Equation (19) has to be replaced by[4]

$$\gamma_{\mathrm{FRET}}=\gamma_{\mathrm{r},0}^{\mathrm{D}}18\pi\int_0^\infty f_0^{\mathrm{D}}(\omega)|\mathbf{n}_{\mathrm{A}}\cdot\mathcal{G}(\mathbf{r}_{\mathrm{A}},\mathbf{r}_{\mathrm{D}},\omega)\cdot\mathbf{n}_{\mathrm{D}}|^2\sigma_{\mathrm{A}}(\omega)\mathrm{d}\omega. \tag{21}$$

Here, $\mathcal{G}(\mathbf{r}_{\mathrm{A}},\mathbf{r}_{\mathrm{D}},\omega)$ is the Green's dyadic that describes the electric field at the position $\mathbf{r}_{\mathrm{A}}$ of the acceptor caused by an oscillating electric dipole at the position $\mathbf{r}_{\mathrm{D}}$ of the donor in the presence of the nanoparticle.

Combination of quenching and FRET

In this section, we show, how the quenching equations were extended in order to include FRET. In general, the fluorescence rates of donor and acceptor are given by Eq. (1), with

$$\gamma_{\mathrm{fl}}^{\mathrm{D}} = q^{\mathrm{D}} \gamma_{\mathrm{exc}}^{\mathrm{D}} \tag{22}$$

and

$$\gamma_{\mathrm{fl}}^{\mathrm{A}} = q^{\mathrm{A}} \gamma_{\mathrm{exc}}^{\mathrm{A}}. \tag{23}$$

In the case of FRET, the term γ_{FRET} has to be included in the quantum yield of the donor, in order to take into account the energy that is transferred to the acceptor. Hence, the quantum yield of the donor becomes

$$q^{\mathrm{D}} = \frac{\gamma_{\mathrm{r}}^{\mathrm{D}}}{\gamma_{\mathrm{r}}^{\mathrm{D}} + \gamma_{\mathrm{abs}}^{\mathrm{D}} + \gamma_{\mathrm{nr}}^{\mathrm{D}} + \gamma_{\mathrm{FRET}}}, \tag{24}$$

with γ_{FRET} given by Eq. (19) or by Eq. (21). Assuming that the acceptor does not absorb at the excitation frequency $\omega_{\mathrm{exc}}^{\mathrm{D}}$ of the donor, the acceptor is only excited via FRET from the donor and its excitation rate becomes

$$\gamma_{\mathrm{exc}}^{\mathrm{A}} = q_{\mathrm{FRET}} \gamma_{\mathrm{exc}}^{\mathrm{D}}, \tag{25}$$

where q_{FRET} is the FRET efficiency defined as

$$q_{\mathrm{FRET}} = \frac{\gamma_{\mathrm{FRET}}}{\gamma_{\mathrm{r}}^{\mathrm{D}} + \gamma_{\mathrm{abs}}^{\mathrm{D}} + \gamma_{\mathrm{nr}}^{\mathrm{D}} + \gamma_{\mathrm{FRET}}}. \tag{26}$$

The quantum yield of the acceptor remains unaffected by the FRET and is

$$q^{\mathrm{A}} = \frac{\gamma_{\mathrm{r}}^{\mathrm{A}}}{\gamma_{\mathrm{r}}^{\mathrm{A}} + \gamma_{\mathrm{abs}}^{\mathrm{A}} + \gamma_{\mathrm{nr}}^{\mathrm{A}}}. \tag{27}$$

Final FRET equations. Using again Eq. (8) to convert the fluorescence rates into spectra and normalizing the resulting spectra to the intrinsic donor peak $F_0^{\mathrm{D}}(\omega_{\mathrm{max}}^{\mathrm{D}})$ in the absence of nanoparticle and acceptor yields the final FRET equations. The emission spectrum of the donor (normalized to the intrinsic donor peak) is then given by

$$\frac{F^{\mathrm{D}}(\omega)}{F_0^{\mathrm{D}}(\omega_{\mathrm{max}})} = \frac{g_{\mathrm{r}}^{\mathrm{D}}(\omega) f_0^{\mathrm{D}}(\omega)}{\int_0^\infty f_0^{\mathrm{D}}(\omega) g_{\mathrm{r}}^{\mathrm{D}}(\omega) \mathrm{d}\omega + \int_0^\infty f_0^{\mathrm{D}}(\omega) g_{\mathrm{abs}}^{\mathrm{D}}(\omega) \mathrm{d}\omega + (1 - q_0^{\mathrm{D}})/q_0^{\mathrm{D}} + \gamma_{\mathrm{FRET}}^{\mathrm{AD}}/\gamma_{\mathrm{r},0}^{\mathrm{D}}} \frac{g_{\mathrm{exc}}^{\mathrm{D}}(\omega_{\mathrm{exc}}^{\mathrm{D}})}{q_0^{\mathrm{D}} f_0^{\mathrm{D}}(\omega_{\mathrm{max}})}. \tag{28}$$

As for the quenching case, here it was assumed that $\hbar\omega_{\mathrm{fl}}^{\mathrm{D}} = \hbar\omega_{\mathrm{fl},0}^{\mathrm{D}}$. The frequency $\omega_{\mathrm{exc}}^{\mathrm{D}}$ denotes the excitation frequency of the donor. The emission spectrum of the acceptor (normalized to the intrinsic donor peak) is

$$\frac{F^{\mathrm{A}}(\omega)}{F_0^{\mathrm{D}}(\omega_{\mathrm{max}})} = \frac{\hbar\omega_{\mathrm{max}}^{\mathrm{A}}}{\hbar\omega_{\mathrm{max}}^{\mathrm{D}}} \frac{g_{\mathrm{r}}^{\mathrm{A}}(\omega) f_0^{\mathrm{A}}(\omega)}{\int_0^\infty f_0^{\mathrm{A}}(\omega) g_{\mathrm{r}}^{\mathrm{A}}(\omega) \mathrm{d}\omega + \int_0^\infty f_0^{\mathrm{A}}(\omega) g_{\mathrm{abs}}^{\mathrm{A}}(\omega) \mathrm{d}\omega + (1 - q_0^{\mathrm{A}})/q_0^{\mathrm{A}}} q_{\mathrm{FRET}} \frac{g_{\mathrm{exc}}^{\mathrm{D}}(\omega_{\mathrm{exc}}^{\mathrm{D}})}{q_0^{\mathrm{D}} f_0^{\mathrm{D}}(\omega_{\mathrm{max}})}. \tag{29}$$

Since the emission frequencies of donor and acceptor are not identical, it cannot be assumed that $\hbar\omega_{\mathrm{fl}}^{\mathrm{A}} = \hbar\omega_{\mathrm{fl},0}^{\mathrm{D}}$. However, it was assumed that the average frequencies of emitted photons can be approximated by the peak frequencies ($\hbar\omega_{\mathrm{fl},0}^{\mathrm{D}} = \hbar\omega_{\mathrm{max}}^{\mathrm{D}}$ and $\hbar\omega_{\mathrm{fl}}^{\mathrm{A}} = \hbar\omega_{\mathrm{max}}^{\mathrm{A}}$), which leads to the factor $\hbar\omega_{\mathrm{max}}^{\mathrm{A}}/\hbar\omega_{\mathrm{max}}^{\mathrm{D}}$. This factor accounts for the fact that the acceptor emits photons of different energies as the donor and the rate equations count the number of photons, while the spectra are a measure for the emitted power. For the actual calculation, the FRET efficiency was rewritten as

$$q_{\mathrm{FRET}} = \frac{\gamma_{\mathrm{FRET}}^{\mathrm{AD}}/\gamma_{\mathrm{r},0}^{\mathrm{D}}}{\int_0^\infty f_0^{\mathrm{D}}(\omega) g_{\mathrm{r}}^{\mathrm{D}}(\omega) \mathrm{d}\omega + \int_0^\infty f_0^{\mathrm{D}}(\omega) g_{\mathrm{abs}}^{\mathrm{D}}(\omega) \mathrm{d}\omega + (1 - q_0^{\mathrm{D}})/q_0^{\mathrm{D}} + \gamma_{\mathrm{FRET}}^{\mathrm{AD}}/\gamma_{\mathrm{r},0}^{\mathrm{D}}}. \tag{30}$$

In these equations, q_0^{A}, $f_0^{\mathrm{A}}(\omega)$, q_0^{D}, and $f_0^{\mathrm{D}}(\omega)$ are intrinsic dye properties, while the quantities $g_{\mathrm{r}}^{\mathrm{A}}(\omega)$, $g_{\mathrm{abs}}^{\mathrm{A}}(\omega)$, $g_{\mathrm{r}}^{\mathrm{D}}(\omega)$, $g_{\mathrm{abs}}^{\mathrm{D}}(\omega)$, and $g_{\mathrm{exc}}^{\mathrm{D}}(\omega_{\mathrm{exc}}^{\mathrm{D}})$ are calculated by numerical simulations. Note that although those last quantities do not directly depend on the intrinsic dye properties, they do depend on the position and orientation of the dyes and are hence also labeled with A and D in order to avoid confusion. The quantity $\gamma_{\mathrm{FRET}}^{\mathrm{AD}}/\gamma_{\mathrm{r},0}^{\mathrm{D}}$ can either be calculated from Eq. (21) using numerical simulations or analytically from Eq. (19). In both cases, the quantity depends on the intrinsic dye properties as well as on the positions and the orientations of donor and acceptor.

Note that the model can be extended in order to include collision quenching[5] by adding a collision quenching rate γ_{col} in the denominators of Eq. (24), Eq. (26), and Eq. (27), which is, however, beyond the scope of this work.

Simulation details

Dye properties used in calculations

The dye properties necessary for the calculation are the intrinsic quantum yields q_0^{D} and q_0^{A}, the normalized intrinsic fluorescence spectra $f_0^{\mathrm{D}}(\omega)$ and $f_0^{\mathrm{A}}(\omega)$, and the absorption cross section of the acceptor $\sigma_{\mathrm{A}}(\omega)$. The quantum yields given by the supplier of the dyes are $q_0^{\mathrm{D}} = 0.8$ and $q_0^{\mathrm{A}} = 0.65$. The intrinsic fluorescence spectra of the dyes (not normalized) are depicted in Supplementary Figure 9(**a**). They were measured for the dyes attached to the DNA origami structure, in order to include possible influences of the DNA on the fluorescence spectrum. The peak wavelengths are 578 nm for the donor and 663 nm for the acceptor. In a similar way, the absorption cross sections were obtained: First, the excitation spectra of the dyes attached to the DNA origami structure were measured, which yielded the spectral shape of the absorption cross sections, but not their absolute values. Then, in order to get the absolute values (given in m^2), the measured spectra were scaled using the maximum molar attenuation coefficients given by the supplier as $\varepsilon_{\mathrm{max}}^{\mathrm{D}} = 1.2 \times 10^5\ \mathrm{M}^{-1}\mathrm{cm}^{-1}$ and $\varepsilon_{\mathrm{max}}^{\mathrm{A}} = 1.5 \times 10^5\ \mathrm{M}^{-1}\mathrm{cm}^{-1}$. Here, it was assumed that the excitation spectra of the dyes are identical to their absorption spectra and that the influence of the DNA on the absolute height of the absorption peaks can be neglected. The absorption cross sections that are obtained by this method are depicted in Supplementary Figure 9(**b**). Note that the absorption cross section of the donor is actually not necessary for the calculations and is only plotted for the sake of completeness.

Results of quenching calculations

The quantities $g_{\mathrm{abs}}(\omega)$, $g_{\mathrm{r}}(\omega)$, and $g_{\mathrm{exc}}(\omega_{\mathrm{exc}}^{\mathrm{x}})$ were derived from finite element simulations using the commercial software COMSOL Multiphysics. The nanoparticles were modeled as gold spheres of diameter 10 nm ($\varepsilon_{\mathrm{Au}}$ from Ref. [6]) embedded into water ($\varepsilon_{\mathrm{water}} = 1.332^2$). The actual sliders contain two nanoparticles, which are placed at a distance of 28 nm. In the simulations, it was found that the influence of the second nanoparticle on the decay rates is negligibly small and only the nanoparticle close to the dye is important. Hence, in order to keep our final model simple, only this nanoparticle was included. Supplementary Figure 10(**a**) shows the simulation results for $g_{\mathrm{r}}(\omega)$ and Supplementary Figure 10(**b**) the simulation results for $g_{\mathrm{abs}}(\omega)$, both at a distance of 5 nm between the dye and the nanoparticle, plotted for two orthogonal dipole orientations. It can be seen that the enhancement factor for the radiative rate has a dip or a peak at around 550 nm, depending on the orientation of the dipole, while the energy transfer factor has a peak at 530 nm for both dipole orientations.

The excitation enhancement factor $g_{\mathrm{exc}}(\omega_{\mathrm{exc}}^{\mathrm{x}})$ was derived from numerical simulations of the electric near-field distribution around the nanoparticle for an incident plane wave. Note that $g_{\mathrm{exc}}(\omega_{\mathrm{exc}}^{\mathrm{x}})$ does not only depend on the distance to the nanoparticle, but also on the relative position of the dye with respect to the direction of the incident field. Since in the experiment, the orientation of DNA sliders is randomly distributed, $g_{\mathrm{exc}}(\omega_{\mathrm{exc}}^{\mathrm{x}})$ was averaged over a spherical surface around the nanoparticle for each distance of dye and nanoparticle. It was found that the averaged $g_{\mathrm{exc}}(\omega_{\mathrm{exc}}^{\mathrm{x}})$ only starts to differ from 1 for distances smaller than 10 nm for the excitation wavelengths used in the experiment (single dye experiments: 530 nm for the donor and 600 nm for the acceptor, FRET experiments: 530 nm).

The quantities $g_{\mathrm{abs}}(\omega)$, $g_{\mathrm{r}}(\omega)$, and $g_{\mathrm{exc}}(\omega_{\mathrm{exc}}^{\mathrm{x}})$ depend on the orientation of the dyes. It was assumed that the dyes can rotate on the DNA linker and that their orientation is random. In order to take this into account, the calculated spectra were averaged over different dye orientations, assuming an equal distribution. Supplementary Figure 11(**a**) shows the results of the quenching simulations, averaged over different dye orientations. It contains the height of the donor and acceptor peak as a function of distance between the dye and the nanoparticle. The curves are normalized to the peaks of the intrinsic dye spectra in the absence of the nanoparticle. The peak wavelengths are 578 nm for the donor and 663 nm for the acceptor. It was found that the interaction with the nanoparticle does not shift the peak wavelengths. In the plot, the excitation enhancement is neglected, in order to allow a comparison of both dyes without the need to take into account that donor and acceptor are usually excited at different wavelengths.

Extracting donor and acceptor contribution from experimental data

The experimental curves in Fig. 3b show the emission intensity of the acceptor at the acceptor peak wavelength $\tilde{F}^{\mathrm{A}}(663\ \mathrm{nm})$ and the emission intensity of the donor at the donor peak wavelength $\tilde{F}^{\mathrm{D}}(578\ \mathrm{nm})$ as a function of time (the tilde is used to indicate that the experimental spectra here are measured in units of wavelength*). The two curves $\tilde{F}^{\mathrm{A}}(663\ \mathrm{nm})$ and $\tilde{F}^{\mathrm{D}}(578\ \mathrm{nm})$ were extracted from emission intensities of the sample at those two

*The experimental spectra in this section are measured in the units of wavelength, while the calculations presented in section **Equations** rely on spectra that are given in units of frequency. The representation $\tilde{F}(\lambda)$ of a spectrum in the sense of a spectral density over wavelength and its representation $F(\omega)$ over frequency are linked by the transformation $\tilde{F}(\lambda) = -\omega^2/(2\pi c)F(\omega)$ (cf. Ref. [7]). The extraction method discussed here can also be applied to spectra given in the units of frequency. However, in this case, it has to be considered that the factor r^{D} used in Eq. (33) takes different values, depending on whether the spectrum is given in units of frequency or in units of wavelength.

wavelengths, denoted as $\tilde{F}^{\text{mes}}(663\text{ nm})$ and $\tilde{F}^{\text{mes}}(578\text{ nm})$ (note that the sliders in the measured sample contain both donors and acceptors). One problem in the evaluation of this measurement was that the emission spectrum of the donor overlaps with the emission spectrum of the acceptor, as it can be seen in Supplementary Figure 9. This means that the spectrum $\tilde{F}^{\text{mes}}(\lambda)$ emitted by the sample is the sum of the spectrum emitted by the donors $\tilde{F}^{\text{D}}(\lambda)$ and the spectrum emitted by the acceptors $\tilde{F}^{\text{A}}(\lambda)$. At the peak wavelengths, this reads as

$$\tilde{F}^{\text{mes}}(663\text{ nm}) = \tilde{F}^{\text{D}}(663\text{ nm}) + \tilde{F}^{\text{A}}(663\text{ nm}) \tag{31}$$

and

$$\tilde{F}^{\text{mes}}(578\text{ nm}) = \tilde{F}^{\text{D}}(578\text{ nm}) + \tilde{F}^{\text{A}}(578\text{ nm}). \tag{32}$$

In order to obtain the curves depicted in Fig. 3b, it was necessary to extract $\tilde{F}^{\text{A}}(663\text{ nm})$ and $\tilde{F}^{\text{D}}(578\text{ nm})$ from the measured intensities $\tilde{F}^{\text{mes}}(663\text{ nm})$ and $\tilde{F}^{\text{mes}}(578\text{ nm})$. In the following, we present the method that was used to extract those contributions and we furthermore show that this method is justified by our simulations.

From measurements of the intrinsic dye spectra, we know that there is almost no emission from the acceptor at the donor peak wavelength, which gives $\tilde{F}^{\text{A}}(578\text{ nm}) \approx 0$ [Note, however, this part of the acceptor spectrum is not plotted in Supplementary Figure 9(**a**)]. From this, it follows that $\tilde{F}^{\text{D}}(578\text{ nm}) \approx \tilde{F}^{\text{mes}}(578\text{ nm})$. At the acceptor peak wavelength, the situation is different. There is a contribution of both donor and acceptor. If the shape of the donor spectrum is known, the donor contribution at the acceptor peak wavelength can be subtracted from the combined intensity $\tilde{F}^{\text{mes}}(663\text{ nm})$. This is done using

$$\tilde{F}^{\text{A}}(663\text{ nm}) = \tilde{F}^{\text{mes}}(663\text{ nm}) - r^{\text{D}}\tilde{F}^{\text{D}}(578\text{ nm}), \tag{33}$$

with

$$r^{\text{D}} = \frac{\tilde{F}^{\text{D}}(663\text{ nm})}{\tilde{F}^{\text{D}}(578\text{ nm})}. \tag{34}$$

The factor r^{D} is defined as the ratio of the donor intensity at the acceptor peak wavelength $\tilde{F}^{\text{D}}(663\text{ nm})$ and the donor intensity at the donor peak wavelength $\tilde{F}^{\text{D}}(578\text{ nm})$. The donor spectrum in Supplementary Figure 9(**a**) yields an intrinsic value of $r^{\text{D}} = 0.106$. However, r^{D} is in general not a fixed number, since – as shown in Eq. (17) – the nanoparticle influences the shape of the donor's emission spectrum. In order to figure out if this effect is relevant for our system, we calculated the dependence of r^{D} on the distance between the donor and the nanoparticle. The result is plotted in Supplementary Figure 11(**b**). It can be seen that for distances larger than 5 nm, r^{D} is constant and takes the intrinsic value $r^{\text{D}} = 0.106$. Only for very small distances, r^{D} starts to decrease. This decrease is caused by the fact that the peak wavelength of the donor (578 nm) experiences a larger emission enhancement $g_{\text{r}}(\omega)$ than the peak wavelength of the acceptor (663 nm), since it is closer to the plasmon resonance (between 530 nm and 550 nm). From the fact that r^{D} is only different from its intrinsic value for distances smaller than 5 nm, and since at these distances the donor intensity should be close to zero anyway [cf. Supplementary Figure 11(**a**)], we conclude, that we can use Eq. (33) together with the intrinsic value $r^{\text{D}} = 0.106$ in order to extract $\tilde{F}^{\text{A}}(663\text{ nm})$ for all sliding states.

It has to be mentioned that in the experiment, the donor peak never reaches zero, even not when the sliders are in a state where the donor is expected to be very close to the nanoparticle; however, this does not limit the applicability of Eq. (33). The nonzero donor signal can be explained by the fact that there is a small fraction of sliders that do not slide and are stuck in some random state. Hence, there are always some donors that are not close to the nanoparticle and emit a background spectrum. Since the donors that cause this background spectrum are not close to the nanoparticle (otherwise they would be quenched), the assumption $r^{\text{D}} = 0.106$ is still valid for those donors and Eq. (33) is still applicable.

Dye positions

The FRET curves depicted in Fig. 3c were calculated using Eq. (28), Eq. (29), and Eq. (30). For the calculation, it was necessary to specify the positions of the dyes during the individual sliding states that are depicted in Fig. 3a.

As shown in Fig. 1 and Fig. 2, the DNA slider consists of two parallel DNA origami filaments with a distance of 12 nm. Based on this value and the geometry of the structure in state '0', we assume that the dyes are bound to single stranded DNA with a length of 6 nm. The location at which the single DNA strands are attached to the DNA origami filaments is explained in Fig. 1. Since those single DNA strands are highly flexible, there is an uncertainty of the dye position. The single stranded DNA has a length of 26 nucleotides. In order to keep the model simple, it is

assumed that the strands are standing perpendicular to the origami filaments. This configuration will be henceforth referred to as unbound state.

When the two dyes come close to each other, the DNA strands can stick together via a transient binding and form a bound state. In this transient binding, the expected distance of the two strands is about 2 nm. However; since the dyes do actually not sit directly at the end of the single DNA strands, but are connected to them by linkers of estimated length 2 nm, there is an additional source for position uncertainty. It was assumed that the effective distance between the donor and the acceptor in the bound state is about 5 nm.

As shown in Fig. 3a, the DNA slider has 5 different sliding states. Supplementary Figure 12 depicts the dye positions that were used in the simulation to represent the individual sliding states. In order to quantitatively describe the positions, a coordinate system is depicted, with the origin corresponding to the center of the nanoparticle (diameter 10 nm). The coordinates can be found in Supplementary Table 2, together with the corresponding distance between the dye and the nanoparticle (labeled as Dye-AuNC) and the corresponding distance between the two dyes (labeled as D-A).

In the '-2' and '2' sliding states, the dyes are very far apart, so they cannot form a bound state. Hence, those sliding states were modeled as an unbound state with the dyes standing perpendicular to the origami filaments. In the '0' sliding state, the dyes are very close to each other and a transient binding is highly likely. Hence, this sliding state was modeled as a bound state with a distance of 5 nm, as explained above. The '-1' and '1' sliding states are slightly more complicated: Due to the intermediate distance, it is expected to be possible that the dyes can be in a bound state as well as in an unbound state. Note that in the '-1' and '1' state, the sliding displacement is 14 nm and geometrical considerations would hence suggest that the single DNA strands with expected length of 6 nm are not long enough to form a bound state. However, it is known that the single DNA strands are stretchable and can in fact be longer than 6 nm. In the simulation, we found, that neither the assumption that all dyes are in an unbound state, nor the assumption that all dyes are in a bound state can explain the experimental results. For an unbound state, with the DNA strands standing perpendicular to the filaments, the distance between the donor and the acceptor is 14 nm, which is much larger than the Förster radius. In this case, the calculated acceptor intensity is close to zero. For a bound state, the calculation yields an acceptor intensity that is comparable to the one in the '0' sliding state (only slightly altered by the different quenching of donor and acceptor compared to the '0' sliding state). In the experiment, the acceptor intensity in the '-1' and '1' sliding states is in between the one from the '0' and the one from the -2' and '2' sliding states. For this reason, it was concluded that there is a combination of dye pairs in a bound state and dye pairs in an unbound state. Since no exact ratio is known, it was assumed that both states are equally likely (50%:50% distribution).

Details on FRET calculations

In order to obtain the curves depicted in Fig.3c, it was necessary to calculate the normalized FRET rates $\gamma_{\mathrm{FRET}}^{\mathrm{AD}}/\gamma_{\mathrm{r,0}}^{\mathrm{D}}$ for the dye configurations occurring in the different sliding states. The normalized FRET rate can be either calculated with Eq. (19), which neglects the influence of the nanoparticle, or with Eq. (21), which takes into account the nanoparticle. For Eq. (19), we used the measured dye properties to calculate the Förster radius, which yields $R_0 = 6.41$ nm (for $\kappa^2 = 2/3$ as the average value). For Eq. (21), we used the measured dye properties as well; however, instead of calculating the Förster radius, we used the local electric field obtained from finite element simulations in order to construct the Green's dyadic. We found that for all donor-acceptor configurations that are relevant in our system, the results of both equations agree extremely well. This can be explained by the fact that in the vicinity of the donor, the magnitude of the initial electric field caused by the donor dipole is is much stronger than the magnitude of the scattered field generated by the nanoparticle, so that the scattered field of the nanoparticle can be neglected. Only in cases where both donor and acceptor are very close to the nanoparticle's surface (in the order of 5 nm), the scattered field becomes relevant and the results start to differ. This agrees with results reported in Ref. [8]. In order to keep the final model simple, the curves depicted in Fig. 3c have been calculated using Eq. (19), neglecting the influence of the nanoparticle on the FRET rate.

The FRET process depends on the polarization of both involved dyes. As for the quenching calculations, it was assumed that the dyes can rotate on the DNA linker and that their orientations are randomly distributed. In order to take this into account, the calculated spectra were averaged over different donor and acceptor orientations simultaneously.

Supplementary References

1. Ringler, M., Schwemer, A., Wunderlich, M., Nichtl, A., Kürzinger, K., Klar, T. A. & Feldmann, J. Shaping emission spectra of fluorescent molecules with single plasmonic nanoresonators. *Phys. Rev. Lett.* **100**, 203002 (2008).

2. Bharadwaj, P. & Novotny, L. Spectral dependence of single molecule fluorescence enhancement. *Opt. Express* **15**, 14266–14274 (2007).

3. Novotny, L. & Hecht, B. *Principles of Nano-Optics* (Cambridge University Press, 2012).

4. Marocico, C. A., Zhang, X. & Bradley, A. L. A theoretical investigation of the influence of gold nanosphere size on the decay and energy transfer rates and efficiencies of quantum emitters. *J. Chem. Phys.* **144**, 024108 (2016).

5. Funke, J. J. & Dietz, H. Placing molecules with bohr radius resolution using dna origami. *Nat. Nanotechnol.* **11**, 47 (2016).

6. Rakić, A. D., Djurišić, A. B., Elazar, J. M. & Majewski, M. L. Optical properties of metallic films for vertical-cavity optoelectronic devices. *Appl. Opt.* **37**, 5271–5283 (1998).

7. Mooney, J. & Kambhampati, P. Get the basics right: Jacobian conversion of wavelength and energy scales for quantitative analysis of emission spectra. *The J. Phys. Chem. Lett.* **4**, 3316–3318 (2013).

8. Gonzaga-Galeana, J. A. & Zurita-Sánchez, J. R. A revisitation of the förster energy transfer near a metallic spherical nanoparticle: (1) efficiency enhancement or reduction? (2) the control of the förster radius of the unbounded medium. (3) the impact of the local density of states. *J. Chem. Phys.* **139**, 244302 (2013).
