# Supplementary material for: Gold nanocrystal-mediated sliding of doublet DNA origami filaments
Source: Nat Commun. 2018 Apr 13;9:1454. doi: 10.1038/s41467-018-03882-w (PMC5899135; doi:10.1038/s41467-018-03882-w)
Supplement: Supplementary file 2 — Description of Additional Supplementary Files [file 41467_2018_3882_MOESM2_ESM.pdf]

Description of Additional Supplementary Files

File Name: Supplementary Data 1
Description: Detailed sequence information of all the DNA strands.
